# Comparison between the NUTRIC score and modified NUTRIC score to predict hospital mortality in patients undergoing cardiac surgery: A retrospective study

**DOI:** 10.1002/ncp.11306

**Published:** 2025-05-05

**Authors:** Jing Xu, Dandong Luo, Ruibin Chi, Jia Deng, Heng Fang, Qingrui Wu, Wang Xu, Jianyang Huang, Chunbo Chen

**Affiliations:** ^1^ Department of Critical Care Medicine Shenzhen People's Hospital (The Second Clinical Medical College, Jinan University; The First Affiliated Hospital, Southern University of Science and Technology) Shenzhen Guangdong China; ^2^ Department of Emergency Maoming People's Hospital Maoming China; ^3^ Department of Intensive Care Unit of Cardiovascular Surgery, Guangdong Provincial People's Hospital (Guangdong Academy of Medical Sciences), Southern Medical University Guangzhou China; ^4^ Department of Critical Care Medicine Xiaolan People's Hospital of Zhongshan Zhongshan Guangdong China; ^5^ Department of Critical Care Medicine Guangdong Provincial People's Hospital (Guangdong Academy of Medical Sciences), Southern Medical University Guangzhou China

**Keywords:** cardiac surgery, hospital mortality, modified NUTRIC score, nutrition risk, NUTRIC score

## Abstract

**Background:**

Nutrition status evaluation is essential for patients undergoing cardiac surgery. The Nutrition Risk in the Critically Ill (NUTRIC) and modified NUTRIC (mNUTRIC) scores are nutrition risk assessment tools specifically for patients in the intensive care unit (ICU). The objective of this study was to validate and compare the accuracy of these two nutrition scores in predicting hospital mortality in patients undergoing cardiac surgery.

**Methods:**

This retrospective study screened adult patients undergoing cardiopulmonary bypass cardiac surgery in the ICU from June 2020 to August 2022. Patients were grouped according to NUTRIC score and mNUTRIC score within 24 h of ICU admission. Logistic regression was used to analyze the risk factors affecting the prognosis of these patients. The area under the receiver operating characteristic curve (AUC‐ROC) was used to compare the predictive performance of these two nutrition scores for hospital mortality.

**Results:**

Data from 252 eligible patients (55.6% of whom were male) were analyzed. It was found that Acute Physiological and Chronic Health Evaluation Ⅱ score, aortic surgery, serum albumin level, NUTRIC score, and mNUTRIC score were independent influencing factors of hospital mortality. The AUC‐ROC of the NUTRIC score and the mNUTRIC score for predicting hospital mortality were 0.830 (95% confidence interval [CI]: 0.778–0.874) and 0.824 (95% CI: 0.771–0.869), respectively. There was no significant difference in ROC curves between the two scores (*P* = 0.492).

**Conclusions:**

Both the NUTRIC and mNUTRIC scores showed good predictive performance for hospital mortality in patients undergoing cardiac surgery, and the mNUTRIC score might be a more convenient and cost‐effective tool for nutrition risk assessment.

**Abbreviations**
APACHE II: Acute Physiological and Chronic Health Evaluation II
AUC‐ROC: area under the receiver operating characteristic curve
BMI: body mass index
CABG: coronary artery bypass grafting
CPB: cardiopulmonary bypass
ICU: intensive care unit
IL‐6: interleukin‐6
IQR: interquartile range
mNUTRIC: modified nutrition risk in the critically ill
MV: mechanical ventilation
NRS‐2002: Nutrition Risk Screening‐2002
NUTRIC: nutrition risk in the critically ill
RRT: renal replacement therapy
SD: standard deviation
SOFA: sequential organ failure assessment

## BACKGROUND

Nutrition status evaluation is crucial for patients undergoing cardiac surgery. Malnutrition is a strong independent risk factor for negative postoperative outcomes and is associated with increased postoperative mortality and morbidity.^1^, ^2^, ^3^, ^4^, ^5^ With the increase in human life expectancy and the improvement of surgical techniques in recent decades, patients undergoing cardiac surgery are now older and have more comorbidities, so they are more likely to suffer from malnutrition.^6^, ^7^, ^8^ Therefore, identifying patients undergoing cardiac surgery at high risk of malnutrition and providing adequate nutrition support are critical.

Guidelines from the Society of Critical Care Medicine (SCCM) and American Society for Parenteral and Enteral Nutrition (ASPEN) recommend that all hospitalized patients need to undergo an initial nutrition screening within 48 h of admission, and a comprehensive nutritional assessment should be performed for patients who are critically ill.^9^ Although a number of screening and assessment tools have been developed and successfully used to assess patient nutrition status, only the Nutrition Risk Screening 2002 (NRS‐2002) and the Nutrition Risk in the Critically Ill (NUTRIC) score are recommended for assessing nutrition risk in patients who are critically ill by the SCCM/ASPEN guidelines because they both evaluate baseline nutrition status and disease severity simultaneously.^9^, ^10^, ^11^, ^12^ However, in a consensus statement of an International Multidisciplinary Expert Group on Cardiac Surgery Nutrition, it was emphasized that the NRS‐2002 must be interpreted with caution because the majority of patients undergoing cardiac surgery have an Acute Physiological and Chronic Health Evaluation (APACHE) II score >10, indicating that these patients are already at high risk for malnutrition (NRS‐2002 ≥3).^11^, ^13^

In contrast, the NUTRIC score, developed by Heyland et al specifically to identify nutrition risk in patients who are critically ill, not only included the APACHE II score but also the sequential organ failure assessment (SOFA) score, number of comorbidities, days from hospital to intensive care unit (ICU) admission, and serum interleukin‐6 (IL‐6) levels.^10^ This score helps to identify patients at high risk of malnutrition and distinguish which patients are most likely to benefit from aggressive nutrition interventions in the ICU. However, serum IL‐6 levels are not routinely measured in critical care settings, which limits the use of the NUTRIC score in clinical practice. Rahman et al developed a modified NUTRIC score (mNUTRIC score) by excluding serum IL‐6 values and demonstrated that the modified score did not affect the accuracy of the NUTRIC score in assessing nutrition status in patients who are critically ill.^14^ Several studies have verified that the NUTRIC score and the mNUTRIC score could be used as effective tools for nutrition risk assessment as well as prognosis prediction.^15^, ^16^, ^17^, ^18^, ^19^ Unfortunately, these scores have not been validated in patients undergoing cardiac surgery.

Therefore, the objective of this retrospective study was to validate and compare the accuracy of the NUTRIC score and the mNUTRIC score in predicting hospital mortality in patients undergoing cardiac surgery.

## METHODS

This retrospective study was conducted using clinical data from the Department of Critical Care Medicine, Maoming People's Hospital Affiliated with Southern Medical University in patients admitted from June 2020 to August 2022. Adult patients (≥18 years old) admitted to the ICU after cardiac surgery with cardiopulmonary bypass were enrolled. Patients undergoing catheter valve, aortic arch, or descending aortic surgery were excluded. All data analyzed (including serum IL‐6) were routine items in the diagnosis and treatment of patients in this hospital. Patient anonymity was ensured. Therefore, informed consent for this study was exempted. The study followed the Declaration of Helsinki and was approved by the Ethics Committee of Maoming People's Hospital (Approval No.: PJ2022MI‐K020‐01).

### Data collection

Patient baseline characteristics and postoperative outcomes were retrieved and recorded from the hospital's electronic medical record system, including age, sex, body mass index (BMI), APACHE Ⅱ score on admission to ICU, comorbidities, type of operation, cardiopulmonary bypass time, aortic cross‐clamp time, intraoperative blood loss, serum albumin level, blood lymphocyte count and hemoglobin within 24 h after ICU admission, renal replacement therapy (RRT), duration of mechanical ventilation (MV), length of stay in the ICU and in‐hospital, and hospital mortality. The types of cardiac surgery included coronary artery bypass grafting, single or combined valve replacement or repair, coronary artery bypass grafting combined valve surgery, and aortic replacement. Hospital mortality was calculated based on the patient's clinical condition at discharge. Each patient's data were included only for the first postoperative admission, even if there were multiple ICU admissions after cardiac surgery. All included data were reviewed and validated by ICU physicians and nurses.

Patients were grouped according to NUTRIC score and mNUTRIC score within 24 h of ICU admission. The NUTRIC score (0–10 points) classified patients according to the sum of scores on the following items: age (0–2 points), APACHE Ⅱ score (0–3 points), SOFA score (0–2 points), comorbidities (0–1 points), length of stay before ICU admission (0–1 points), and serum IL‐6 (0–1 points). The mNUTRIC score, which excludes serum IL‐6, ranges from 0 to 9. An NUTRIC score ≥6 and mNUTRIC score ≥5 were considered to indicate a high risk for malnutrition.^10^, ^14^

### Statistical analysis

Continuous variables were described as the mean ± standard deviation (SD) or median (interquartile range [IQR]) based on normality, and categorical variables were described as numbers (and percentage). The student *t* test or the Mann‒Whitney *U* test was used to compare continuous variables, whereas the *χ* ^2^ test or Fisher exact test was used to compare categorical variables. Logistic regression was used to analyze the risk factors affecting the prognosis of patients undergoing cardiac surgery. The discrimination for predicting hospital mortality between the NUTRIC score and the mNUTRIC score was assessed by the area under the receiver operating characteristic curve (AUC‐ROC), and the Youden index was applied to realize the threshold for high nutrition risk. The differences in ROC curves were conducted using MedCalc statistical software (version 20.0.3; MedCalc Software). All other statistical analyses were performed with SPSS Software (version 21.0; SPSS Inc). A two‐sided *P* value < 0.05 was considered as statistically significant.

## RESULTS

### Characteristics of the study participants

A total of 349 patients were screened, of whom 33 were excluded because their age was <18 years and 64 were excluded because of incomplete data. Finally, 252 patients were included in the analysis, and their detailed characteristics are shown in Table 1. The median age was 60 years (IQR, 52.5–67.0), the median BMI was 21.3 (IQR, 19.2–24.1) kg/m^2^, and 140 patients were male (55.6%). The median APACHE Ⅱ score at ICU admission was 16 (IQR, 13–20). The percentage of patients with hypertension and stroke were relatively high, accounting for 30.2% and 19.8%, respectively. The median NUTRIC and mNUTRIC scores were both 4 (IQR, 3–5), and the median serum albumin level was 30.9 g/L (IQR, 27.6–33.9) within 24 h after ICU admission. The hospital mortality of patients after cardiac surgery was 15.9%.

**Table 1:** Characteristics of patients undergoing cardiac surgery.

Characteristics	All patients (*n* = 252)
Age, median (minimum to maximum), years	60 (52.5–67.0)
Male, *n* (%)	140 (55.6)
BMI, median, (minimum to maximum), kg/m^2^	21.3 (19.2–24.1)
APACHE Ⅱ score, median, (minimum to maximum)	16 (13–20)
Comorbidities	
Hypertension, *n* (%)	76 (30.2)
Diabetes, *n* (%)	28 (11.1)
Chronic kidney disease, *n* (%)	11 (4.4)
Stroke, *n* (%)	50 (19.8)
Operative variables	
CABG alone, *n* (%)	23 (9.1)
Valve alone, *n* (%)	178 (70.6)
CABG and valve surgery, *n* (%)	26 (10.3)
Aortic surgery, *n* (%)	25 (9.9)
CPB time, median, (minimum to maximum), minutes	192 (150.0–259.5)
Cross‐clamp time, median, (minimum to maximum), minutes	148 (113.0–200.5)
Blood loss, median, (minimum to maximum), ml	300 (300–400)
Variables within 24 h of ICU admission	
NUTRIC score, median, (minimum to maximum)	4 (3–5)
mNUTRIC score, median, (minimum to maximum)	4 (3–5)
Serum total protein, median, (minimum to maximum), g/L	56.7 (50.8–60.8)
Serum albumin level, median, (minimum to maximum), g/L	30.9 (27.6–33.9)
Blood lymphocyte count, median, (minimum to maximum), 10^9^/L	1.1 (0.7–1.7)
Hemoglobin, median, (minimum to maximum), g/L	108 (94.5–121.5)
Postoperative outcomes	
RRT, *n* (%)	15 (6.0)
Duration of MV, median, (minimum to maximum), days	1 (1–2)
Length of stay in ICU, median, (minimum to maximum), days	1 (1–2.5)
Length of stay in hospital, median, (minimum to maximum), days	30.5 (25–38)
Hospital mortality, *n* (%)	40 (15.9)

*Note*: Quantitative variables were expressed as the median (IQR) and qualitative variables as number (percentage, %).

Abbreviations: APACHE, acute physiology and chronic health evaluation; BMI, body mass index; CABG, coronary artery bypass grafting; CPB, cardiopulmonary bypass; ICU, intensive care unit; IQR, interquartile range; mNUTRIC, modified nutrition risk in the critically ill; MV, mechanical ventilation;NUTRIC, nutrition risk in the critically ill; RRT, renal replacement therapy.

### Comparison of characteristics in patients with different nutrition risks

All patients were divided into high and low nutrition risk groups according to the NUTRIC score and the mNUTRIC score within 24 h after ICU admission, respectively. There were 57 patients with high NUTRIC scores and 82 patients with high mNUTRIC scores. The results of the comparison of baseline characteristics and postoperative outcomes between patients at high and low nutrition risk are presented in Table 2.

**Table 2:** Comparison of characteristics of patients undergoing cardiac surgery in high and low nutrition risk groups.

Variable	NUTRIC score (*n* = 252)			mNUTRIC score (*n* = 252)		
	Low risk (*n* = 195)	High risk (*n* = 57)	*P*	Low risk (*n* = 170)	High risk (*n* = 82)	*P*
Age, median, (minimum to maximum), years	58 (50.5–65.0)	66 (58–73)	<0.001	58 (49–64)	66 (58–73)	<0.001
Male, *n* (%)	102 (52.3)	38 (66.7)	0.055	86 (50.6)	54 (65.9)	0.022
BMI,median, (minimum to maximum), kg/m^2^	21.2 (19.1–24.0)	22 (19.5–24.8)	0.135	21.0 (18.9–23.8)	22 (19.6–25.0)	0.058
APACHE Ⅱ score, median, (minimum to maximum)	15 (12–17)	23 (20–26)	<0.001	15 (12–17)	22 (18–25)	<0.001
Comorbidities						
Hypertension, *n* (%)	47 (24.1)	29 (50.9)	<0.001	36 (21.2)	40 (48.8)	<0.001
Diabetes mellitus, *n* (%)	18 (9.2)	10 (17.5)	0.079	11 (6.5)	17 (20.2)	0.001
Chronic kidney disease, *n* (%)	7 (3.6)	4 (7.0)	0.456	4 (2.4)	7 (8.5)	0.055
Stroke, *n* (%)	28 (14.4)	22 (38.6)	<0.001	19 (11.2)	31 (37.8)	<0.001
Operative variables						
CABG alone, *n* (%)	18 (9.2)	5 (8.8)	0.916	13 (7.6)	10 (12.2)	0.240
Valve alone, *n* (%)	149 (76.4)	29 (50.9)	<0.001	132 (77.6)	46 (56.1)	<0.001
CABG and valve surgery, *n* (%)	15 (7.7)	11 (19.3)	0.011	14 (8.2)	12 (14.6)	0.118
Aortic surgery, *n* (%)	13 (6.7)	12 (21.1)	0.001	11 (6.5)	14 (17.1)	0.008
CPB time, median, (minimum to maximum), minutes	181 (143–222)	267 (192–335)	<0.001	180 (138–217)	242 (173–306)	<0.001
Cross‐clamp time, median, (minimum to maximum), minutes	139 (104–175)	205 (149–233)	<0.001	137 (96–174)	182.5 (131–231)	<0.001
Blood loss, median, (minimum to maximum), ml	300 (275–400)	500 (300–800)	<0.001	300 (220–400)	400 (300–800)	<0.001
Variables within 24 h of ICU admission						
Serum total protein, median, (minimum to maximum), minutes, g/L	58 (52.3–61.3)	52.3 (44.6–56.6)	<0.001	58.4 (52.8–61.3)	52.7 (45.8–58.2)	<0.001
Serum albumin level, median, (minimum to maximum), g/L	31.6 (28.9–34.4)	28(23.9–31.1)	<0.001	31.8 (29.3–34.5)	28.4 (24.3–31.6)	<0.001
Blood lymphocyte count, median, (minimum to maximum), 10^9^/L	1.1 (0.7–1.5)	1.2 (0.8–2.0)	0.095	1.1 (0.6–1.5)	1.2 (0.8–1.9)	0.082
Hemoglobin, median, (minimum to maximum), g/L	111 (100.5–126.0)	94 (83–108)	<0.001	112 (102–127)	96.5 (84–112)	<0.001
Postoperative outcomes						
RRT, *n* (%)	3 (1.5)	12 (21.1)	<0.001	1 (0.6)	14 (17.1)	<0.001
Duration of MV, median, (minimum to maximum), days	1 (1–1)	2 (1–4)	<0.001	1 (1–1)	2 (1–4)	<0.001
Length of stay in ICU, median, (minimum to maximum), days	1 (1–1)	3 (1–5)	<0.001	1 (1–1)	2 (1–5)	<0.001
Length of stay in hospital, median, (minimum to maximum), days	31 (26–37)	30 (15–43)	0.569	30 (26–37)	33 (17–43)	0.671
Hospital mortality, *n* (%)	12 (6.2)	28 (49.1)	<0.001	9 (5.1)	31 (37.8)	<0.001

*Note*: Quantitative variables are expressed as medians (IQR), and qualitative variables are expressed as numbers (percentage, %)

An NUTRIC score ≥6 and mNUTRIC score ≥5 were considered to indicated a high risk for malnutrition. An NUTRIC score ≤5 and mNUTRIC score ≤4 were considered to indicated a low risk for malnutrition.

Abbreviations: APACHE, acute physiology and chronic health evaluation; BMI, body mass index; CABG, coronary artery bypass grafting; CPB, cardiopulmonary bypass; ICU, intensive care unit; IQR, interquartile range; mNUTRIC, modified nutrition risk in critically ill; MV, mechanical ventilation; NUTRIC, nutrition risk in the critically ill; RRT, renal replacement therapy.

In the two nutrition scores, patients in the high‐risk group were older, had more comorbidities, had longer cardiopulmonary bypass time and cross‐clamp time, and had higher APACHE Ⅱ score than those in the low‐risk group (*P* < 0.001). In the comparison of postoperative outcome variables, high‐risk patients had a higher RRT rate, longer ICU stay, longer MV, and higher hospital mortality (*P* < 0.001). No significant differences in length of hospital stay were observed between the high and low nutrition risk group.

### Hospital mortality according to scores

**Figure 1:**
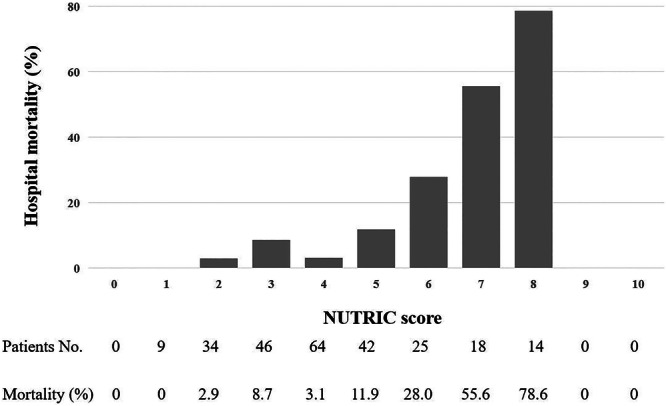
Hospital mortality in patients undergoing cardiac surgery with different Nutrition Risk in the Critically Ill (NUTRIC) scores.

**Figure 2:**
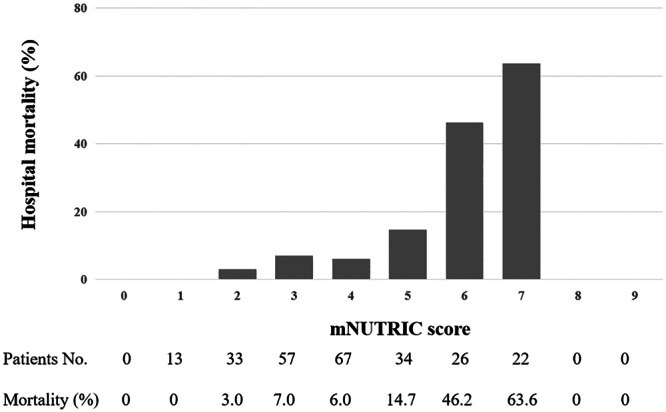
Hospital mortality in patients undergoing cardiac surgery with different modified Nutrition Risk in the Critically Ill (mNUTRIC) scores.

As shown in Figures 1 and 2, the NUTRIC score of our study population ranged from 1 to 8, and the hospital mortality was 78.6% (11 of 14) for patients with the maximum NUTRIC score, whereas the mNUTRIC score ranged from 1 to 7, and the mortality rate of the maximum mNUTRIC score was 63.6% (14/22). Moreover, the two scores had a similar trend; that is, the higher the score, the higher the hospital mortality.

### Logistic regression analysis of prognostic factors in patients undergoing cardiac surgery

Univariate logistic regression analysis was performed on all indicators in Table 1 except postoperative outcomes. It was found that age, BMI, APACHE Ⅱ score, hypertension, stroke, aortic surgery, valve surgery, cardiopulmonary bypass time, cross‐clamp time, intraoperative blood loss, NUTRIC score, mNUTRIC score, serum albumin level, hemoglobin, and lymphocyte count were associated with hospital mortality (*P* < 0.1). The results of multivariate logistic regression analysis of these two nutrition scores were shown in Table 3, and it was found that APACHE Ⅱ score, aortic surgery serum albumin level, NUTRIC score, and mNUTRIC score were independently associated with hospital mortality in patients undergoing cardiac surgery.

**Table 3:** Multivariable logistic regression analyses for hospital mortality of patients undergoing cardiac surgery.

Prediction model and component	OR	95% CI	*P*
Model 1			
APACHE Ⅱ score	1.189	1.043–1.355	0.009
Aortic surgery	58.548	10.876–315.180	<0.001
Serum albumin level	0.938	0.887–0.992	0.026
NUTRIC score	1.784	1.138–2.796	0.012
Model 2			
APACHE Ⅱ score	1.205	1.060–1.370	0.004
Aortic surgery	54.07	10.198–286.665	<0.001
Serum albumin level	0.939	0.889–0.992	0.024
mNUTRIC score	1.802	1.082–3.001	0.024

Abbreviations: APACHE, acute physiology and chronic health evaluation; CI, confidence interval; mNUTRIC, modified nutrition risk in the critically ill; NUTRIC, nutrition risk in the critically ill; OR, odds ratio.

### AUC of scores for predicting hospital mortality

**Figure 3:**
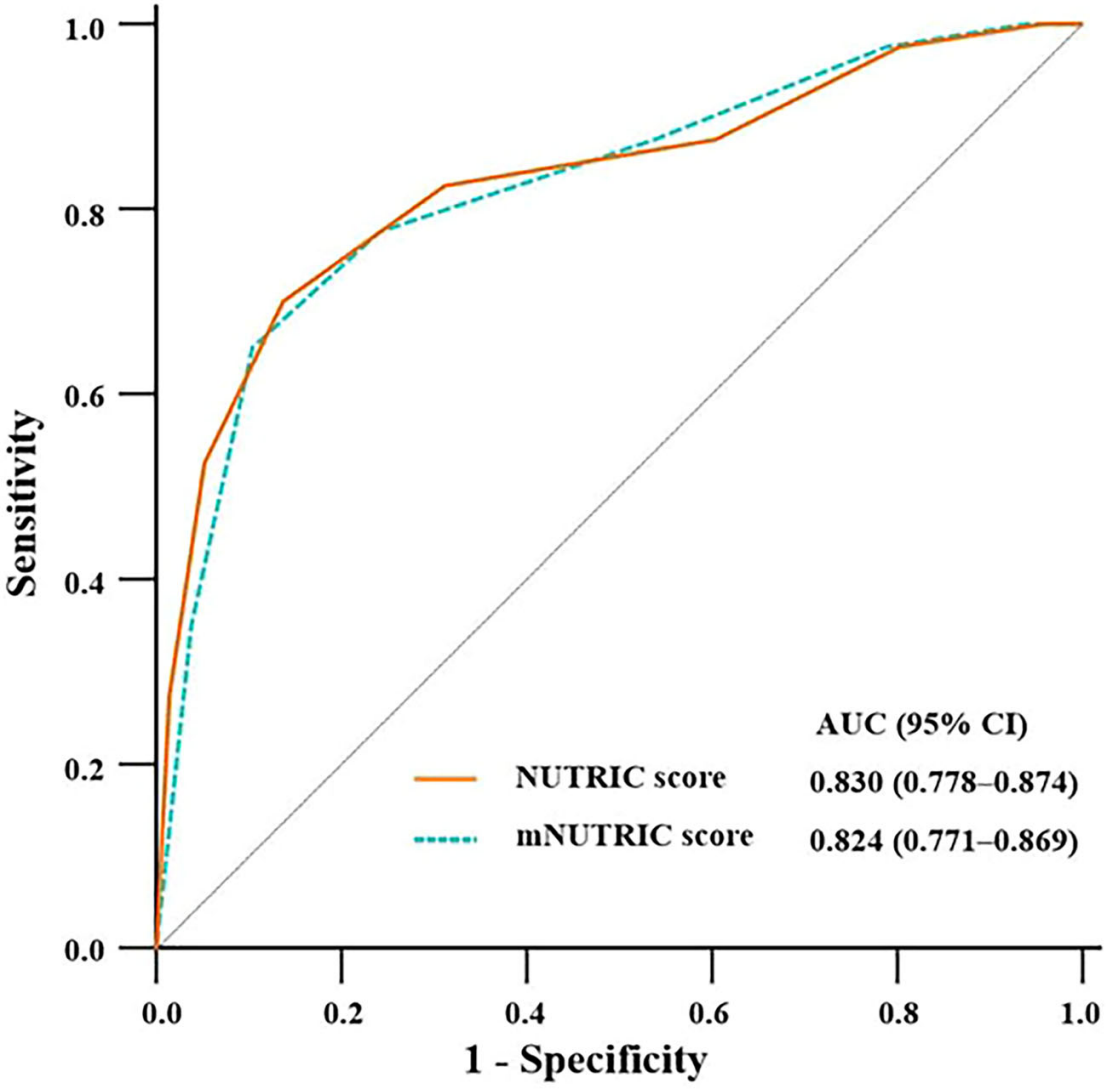
Performance of the Nutrition Risk in the Critically Ill (NUTRIC) score and modified NUTRIC (mNUTRIC) score in predicting hospital mortality in patients undergoing cardiac surgery. There was no significant difference in receiver operating characteristic (ROC) curves between these two scores (*P* = 0.492). The figure is based on a cutoff value of 6 for NUTRIC and mNUTRIC. AUC, area under curve; CI, confidence interval.

As shown in Figure 3 and Table 4, the AUC‐ROC of the NUTRIC score for predicting hospital mortality after cardiac surgery was 0.830 (95%CI: 0.778–0.874), the Jorden index was 0.563, the cutoff criterion was >5, the sensitivity was 70.0%, and the specificity was 86.3%. The AUC‐ROC of the mNUTRIC score for predicting hospital mortality were 0.824 (95% CI: 0.771–0.869). The cutoff criterion was >5; the sensitivity and specificity were 65.0% and 89.6%, respectively; and the Yoden index was 0.546. There was no significant difference in ROC curves between the two scores (*P* = 0.492).

**Table 4:** Performance of the NUTRIC score and mNUTRIC score in predicting hospital mortality in patients undergoing cardiac surgery.

Variable	AUC‐ROC	95% CI	Cutoff value	Sensitivity	Specificity	Youden index
NUTRIC score	0.830	0.778–0.874	6	70.0%	86.3%	0.563
mNUTRIC score	0.824	0.771–0.869	6	65.0%	89.6%	0.546

Abbreviations: AUC‐ROC, area under the receiver operating characteristic curve; CI, confidence interval; mNUTRIC, modified nutrition risk in the critically ill; NUTRIC, nutrition risk in the critically ill.

## DISCUSSION

This retrospective study revealed that both the NUTRIC score and the mNUTRIC score were effective tools for predicting hospital mortality in patients undergoing cardiac surgery, and the NUTRIC score was not superior to the mNUTRIC score. In the comparison of the high and low nutrition risk groups, a high nutrition score was associated with increased RRT rate, prolonged ICU stay and MV, and increased hospital mortality. The AUC‐ROC of the two nutrition scoring systems for predicting hospital mortality was >0.8, and there was no significant difference between the two scores, indicating that both scoring systems had good predictive performance. Because serum IL‐6 levels are currently not routinely measured in the vast majority of ICUs, the mNUTRIC score is considered to be convenient and cost‐effective, conducive to the timely assessment of nutrition risk, and enables more cardiac surgery patients to benefit from aggressive nutrition interventions.

Cardiac surgery is currently an important treatment for cardiovascular diseases, despite the progress made in percutaneous therapy. Patients undergoing cardiac surgery often experience systemic inflammatory responses, and even progress to persistent organ damage.^6^, ^20^, ^21^, ^22^ Improvements in surgical techniques, anesthesia, and perioperative management have minimized the stressful stimulus to catabolism, thus slowing down the energy expenditure process and meeting metabolic demands with fewer nutrients.^23^ Because an increasing number of elderly people require cardiac surgery, comorbidities such as renal insufficiency, diabetes, hypertension and congestive heart failure are increasing.^4^ In this study, the median age of patients was 60 years old, most patients had one or more comorbidities, all patients required MV, and the median APACHE Ⅱ score reached 16. Considering their comorbidities and the severity of the disease, we believe that the current situation of patients undergoing cardiac surgery is of great concern because they are more likely to be at high nutrition risk.

In this study, there were 57 patients with high NUTRIC scores and 82 patients with high mNUTRIC scores. The mNUTRIC scores are 44% higher than the NUTRIC scores. We have fully considered the reasons for this difference. The main difference between the mNUTRIC score and the NUTRIC score is the removal of IL‐6 (1 point). An NUTRIC score of 6 or greater is considered to be at high risk of malnutrition. An mNUTRIC score ≥5 is considered to have a high risk of malnutrition. Therefore, patients with NUTRIC scores ≥6 could be considered high‐risk patients with mNUTRIC scores. In addition, in this study, we also found that the best cutoff of the ROC curve for mNUTRIC score was at 6 (sensitivity of 65.0% and specificity of 89.6%), and the Youden index was 0.546 in this study. However, in another study, the best cutoff was at 5 (sensitivity of 72% and specificity of 63%, respectively), and the Youden index was 0.34.^24^ The simple method of subtracting 1 from the NUTRIC score to find the mNUTRIC score may be inaccurate because the IL‐6 level is excluded from the mNUTRIC score. Further investigation is needed to find the best cutoff score for the high‐risk group in the mNUTRIC score.

Nutrition support was a key component in the perioperative treatment of these patients who are critically ill.^25^ Malnutrition due to inadequate caloric and protein intake during the postoperative period is associated with high morbidity and mortality.^26^, ^27^ Regrettably, a comprehensive analysis of 144 ICU nutrition surveys from around the world found that patients undergoing cardiac surgery were at high risk of inadequate nutrition treatment.^28^ Similarly, an international study found that nutrition support for patients undergoing cardiac surgery was the least adequate compared with other types of surgery.^29^ Clinical physicians have carried out many studies to try to improve this poor nutrition status in a variety of ways, including developing nutrition assessment tools,^30^, ^31^, ^32^ optimizing nutrition support pathways,^33^, ^34^, ^35^, ^36^ and exploring the best combination of nutrients.^37^, ^38^

Considering that nutrition status screening is an essential aspect of good nutrition practice, a series of nutrition risk screening and nutrition assessment tools have been developed for different pathological conditions, such as the Malnutrition Universal Screening Tool,^39^ the NRS‐2002,^11^ the Mini‐Nutritional Assessment,^40^ the Short Nutrition Assessment Questionnaire,^41^ and the Subjective Global Assessment.^42^ Although the aforementioned scoring systems have been validated in a variety of clinical settings, none of these scores have shown sufficient sensitivity or specificity to become the gold standard in the population undergoing cardiac surgery.^2^ Conceptually, an easy‐to‐calculate nutrition score, using variables readily available to cardiac surgeons and intensive care physicians, could help to identify patients who need complementary nutrition support therapy (enteral or parenteral nutrition), effectively reducing the time to start nutrition intervention and benefiting more patients.^30^ The NUTRIC score, which incorporates markers reflecting acute and chronic malnutrition, is an important nutrition scoring system designed specifically for patients who are critically ill to assess the risk of malnutrition and is, additionally, a useful prognostic marker.^10^ In addition, the mNUTRIC score excluding serum IL‐6 has been shown to be a good predictor of morbidity and mortality in postoperative acute care units.^43^ However, no relevant study has yet evaluated and confirmed the important role of the NUTRIC score and mNUTRIC score in the risk assessment of malnutrition and prognostic prediction in patients undergoing cardiac surgery.

In the present study, we screened a total of 252 patients undergoing cardiac surgery to validate and compare the performance of the NUTRIC score and mNUTRIC score in nutrition risk assessment and hospital mortality prediction, hoping to identify patients who might benefit from aggressive nutrition interventions. We grouped patients according to these two nutrition scores and found that high‐risk patients had higher rates of RRT, longer ICU stays, longer MV, and higher hospital mortality. Similarly, in a prospective study, Kalaiselvan et al found that 42.5% of patients receiving MV who were critically ill were at nutrition risk and that high mNUTRIC scores were associated with increased ICU length of stay and higher mortality.^44^ In a systematic review assessing the use of the NUTRIC score around the world, a high NUTRIC score was significantly associated with MV, length of ICU or hospital stay, and mortality.^45^ The results of these studies are consistent, suggesting that patients with high nutrition risk should receive timely nutrition intervention to reduce nutrition‐related complications or mortality.

The NUTRIC score of the study population ranged from 1 to 8, and the mNUTRIC score ranged from 1 to 7. No death occurred in patients with an NUTRIC score or mNUTRIC score of 1. The hospital mortality was 2.9% and 8.7% for patients with NUTRIC scores 2 and 3, whereas 55.6% and 78.6% for patients with NUTRIC scores 7 and 8, respectively. Patients with mNUTRIC scores of 6 and 7 had significantly higher in‐hospital mortality than patients with other mNUTRIC scores. With the increase of nutrition score, the hospital mortality of patients after cardiac surgery had an obvious increasing trend. Moreover, our study confirmed that both the NUTRIC score and mNUTRIC score showed good predictive performance for hospital mortality in patients undergoing cardiac surgery, and the sensitivity of the mNUTRIC score was better. Machado et al also demonstrated the good discriminative power of the mNUTRIC score in quantifying the risk of hospital mortality.^46^ Given that serum IL‐6 measurement is not part of routine hospital care, we believe that the mNUTRIC score is more convenient and economical for the use of clinically available indicators for nutrition risk assessment.

However, this study does have certain limitations. First, this study was retrospective, and the data were collected from a single medical center with a relatively small sample size. Second, the patients included in this study had a short length of ICU stay, which may have some bias because of different surgical methods and patient severity. Third, feeding parameters, such as the route of nutrition support (oral, enteral, or parenteral nutrition) and the adequacy of protein and calorie supplementation, were not collected and evaluated in this study, which prevented our results from confirming an association between nutrition adequacy, nutrition score, and mortality. Therefore, further studies are needed for a comprehensive evaluation to promote the application of the NUTRIC score and mNUTRIC score in patients undergoing cardiac surgery.

## CONCLUSIONS

Both the NUTRIC and mNUTRIC scores could be used as effective tools for nutrition risk screening and hospital mortality prediction in patients undergoing cardiac surgery. Considering that serum IL‐6 measurements are not routinely performed, the mNUTRIC score seems to be more convenient, economical, and conducive to the timely identification of patients with high nutrition risk. More large prospective studies are needed to demonstrate and compare the effectiveness of these scores.

## AUTHOR CONTRIBUTIONS

Chunbo Chen contributed to the conception and design of the research; Jing Xu contributed to the design of the research; Dandong Luo and Ruibin Chi contributed to the analysis and interpretation of the data; Jia Deng, Heng Fang, and Qingrui Wu contributed to the acquisition and analysis of the data; Wang Xu and Jianyang Huang drafted the manuscript; and all authors critically revised the manuscript, agree to be fully accountable for ensuring the integrity and accuracy of the work, and read and approved the final manuscript.

## CONFLICT OF INTEREST STATEMENT

None declared.
